# A Founder Intronic Variant in *P3H1* Likely Results in Aberrant Splicing and Protein Truncation in Patients of Karen Descent with Osteogenesis Imperfecta Type VIII

**DOI:** 10.3390/genes14020322

**Published:** 2023-01-26

**Authors:** Piranit Nik Kantaputra, Salita Angkurawaranon, Worrachet Intachai, Chumpol Ngamphiw, Bjorn Olsen, Sissades Tongsima, Timothy C. Cox, James R. Ketudat Cairns

**Affiliations:** 1 Center of Excellence in Medical Genetics Research, Faculty of Dentistry, Chiang Mai University, Chiang Mai 50200, Thailand; 2 Division of Pediatric Dentistry, Department of Orthodontics and Pediatric Dentistry, Faculty of Dentistry, Chiang Mai University, Chiang Mai 50200, Thailand; 3 Division of Diagnostic Radiology, Department of Radiology, Faculty of Medicine, Chiang Mai University, Chiang Mai 50200, Thailand; 4 National Biobank of Thailand, National Science and Technology Development Agency, Khlong Luang, Pathum Thani 12120, Thailand; 5 Department of Developmental Biology, Harvard School of Dental Medicine, Boston, MA 02115, USA; 6 Departments of Oral & Craniofacial Sciences, School of Dentistry, and Pediatrics, School of Medicine, University of Missouri-Kansas City, Kansas City, MO 64108, USA; 7 Center for Biomolecular Structure, Function and Application and School of Chemistry, Institute of Science, Suranaree University of Technology, Nakhon Ratchasima 30000, Thailand; 8 Laboratory of Biochemistry, Chulabhorn Research Institute, Bangkok 10210, Thailand

**Keywords:** osteogenesis imperfecta type VIII, P3H1 variant, popcorn calcification, brittle bone disease, prolyl 3-hydroxylation complex, Karen tribe

## Abstract

One of the most important steps in post-translational modifications of collagen type I chains is the hydroxylation of carbon-3 of proline residues by prolyl-3-hydroxylase-1 (P3H1). Genetic variants in *P3H1* have been reported to cause autosomal recessive osteogenesis imperfecta (OI) type VIII. Clinical and radiographic examinations, whole-exome sequencing (WES), and bioinformatic analysis were performed in 11 Thai children of Karen descent affected by multiple bone fractures. Clinical and radiographic findings in these patients fit OI type VIII. Phenotypic variability is evident. WES identified an intronic homozygous variant (chr1:43212857A > G; NM_022356.4:c.2055 + 86A > G) in *P3H1* in all patients, with parents in each patient being heterozygous for the variant. This variant is predicted to generate a new “CAG” splice acceptor sequence, resulting in the incorporation of an extra exon that leads to a frameshift in the final exon and subsequent non-functional P3H1 isoform a. Alternative splicing of *P3H1* resulting in the absence of functional P3H1 caused OI type VIII in 11 Thai children of Karen descent. This variant appears to be specific to the Karen population. Our study emphasizes the significance of considering intronic variants.

## 1. Introduction

Osteogenesis imperfecta (OI), a group of hereditary connective tissue disorders, is characterized by brittle bones with increased susceptibility to fractures following minimal trauma, blue sclerae, hyperelastic tendons and ligaments, and growth deficiency. Most cases of OI have autosomal dominant modes of inheritance and are caused by pathogenic variants in either the *COL1A1* [MIM #120150] or *COL1A2* [MIM #120160] gene. *COL1A1* and *COL1A2* encode components of type I collagen, the major protein component of the extracellular matrix in bones, skin, and tendons [1]. Pathogenic variants that result in changes in the quantity of normal collagen generally result in a mild form of OI type I [2]. In contrast, pathogenic variants that lead to changes in the structure of collagen are generally associated with more severe phenotypes.

Collagen type I is a heterotrimer containing two α1 chains and one α2 chain. It is synthesized as a procollagen molecule with a central triple-helical domain lying between N-terminal and C-terminal globular propeptides. The central triple-helical domain is composed of 338 repeats of uninterrupted Gly-X-Y motifs, where X and Y are often proline and 4-hydroxyproline. This arrangement is responsible for the formation of the unique helical tertiary structure. The N- and C-terminal propeptides are ultimately removed extracellularly through cleavage by specific N-terminal and C-terminal propeptidases, a step that is important for the complete maturation of collagen fibers.

Prior to secretion, procollagens go through several steps of post-translational modification in the endoplasmic reticulum (ER) that are important for proper procollagen folding, secretion, and extracellular matrix assembly [3,4]. One of the most important steps is the hydroxylation of carbon-3 of the proline residues α1(I) Pro986 and α2(I) Pro707 by a trimeric collagen-prolyl 3-hydroxylation complex, while the collagen chains are unfolded. The complex contains prolyl-3-hydroxylase-1 (P3H1, encoded by *P3H1*), cartilage-associated protein (encoded by *CRTAP*), and the prolyl cis-trans isomerase cyclophilin-B (CYPB, encoded by *PPIB*). P3H1 plays important roles in the extracellular matrix and the endoplasmic reticulum/Golgi compartments via its RGD (Arg-Gly-Asp) cell adhesion domain and a KDEL (Lys-Asp-Glu-Leu) endoplasmic reticulum (ER) retention sequence, respectively [5,6]. These post-translational modifications of type I procollagen chains are required for the normal folding and stability of the triple helix [5]. The absence of P3H1 leads to the over-modification of type I collagen chains [7]. Genetic variants in *P3H1* and *CRTAP* result in an abnormal 3-hydroxylation complex, post-translational modification defects, and structural abnormalities of collagen and OI types VIII and VII, respectively [5,8,9,10].

Recently, we reported four affected Karen (a hill tribe in Southeast Asia) children with OI type VIII and dental anomalies. The patients were found to carry a homozygous variant in *P3H1* (chr1:43212857A > G; NM_001243246.1; c.2141A > G), which was interpreted as affecting only isoform c of *P3H1* (NP_071751.3; p.Lys714Arg), because the variant falls in the coding region of isoform c but is in a noncoding region for the main isoform, isoform a [11]. However, this analysis conflicts with a previous study that showed that patients in which the mRNA splice form encoding isoform a was eliminated had no detectable P3H1 protein and severely reduced Pro986 3-hydroxylation in the collagen α1(1) chain [12]. These patients appeared to have relatively high levels of transcripts for P3H1 isoform b and isoform c, suggesting that only isoform a is functional. The isoform b transcript contains an extra 19 nucleotides at the end of exon 14, while the isoform c transcript retains intron 14 compared to the isoform a transcript. These differences in isoform b and isoform c shift the reading frame and result in a lack of the C-terminal KDEL ER retention signal. So, it may be expected that isoforms b and c cannot function in the same manner as isoform a to modify the collagen precursor protein in the endoplasmic reticulum.

Here, we report on 11 new patients from seven related families from the Karen population affected by OI type VIII. All patients carried the same homozygous variant (chr1:43212857A > G; NM_022356.4:c.2055 + 86A > G) in the *P3H1* gene as the previously reported Karen patients [11]. However, based on the evidence that isoform a is the primary functional isoform, we have evaluated the effect of this mutation on that isoform. We present evidence that this variant creates a new “CAG” splice acceptor sequence, which is predicted to result in an alternative, novel C-terminal sequence and premature protein truncation. Since all patients, their parents, and relatives are of Karen ancestry and have lived in the same villages for many generations, a founder effect is suspected. This genetic variant appears to be specific to the Karen population.

## 2. Materials and Methods

### 2.1. Patient Recruitment

This study involving human participants was approved by the Human Experimentation Committees of the Faculty of Dentistry, Chiang Mai University (no. 71/2020), and was performed in accordance with the ethical standards of the 1964 Declaration of Helsinki and its later amendments or comparable ethical standards. Informed consent was obtained from all participants.

**Figure 1:**
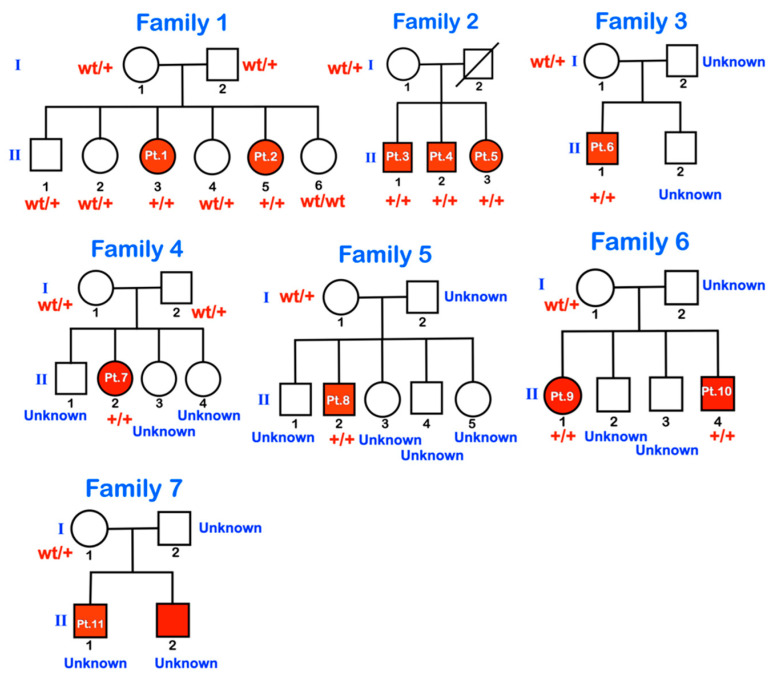
Pedigrees of families 1–7. wt = wild type; +/+ = homozygous for the variant; wt/+ = heterozygous for the variant. Unknown means genetic testing was not performed.

We investigated 11 patients from seven families (Figure 1), all living in Suan Pheurng city, Ratchaburi province, Thailand, near the border of Myanmar. The patients, their parents, and their ancestors belong to the Karen tribe originating in Myanmar. They migrated from Myanmar to their current location about 20 years ago. Very few parents knew whether they were related to each other.

All patients were delivered by midwives at term. Parents reported no history of teratogenic exposure or drug ingestion during pregnancy. The information regarding the pregnancy was minimal. The families were poor and not well educated. Parents were unable to provide information on the medical histories of their children. All affected patients had white sclerae and a short barrel-shaped chest. Ten patients (patients 1–9 and 11) were available for clinical and radiographic examinations. Patient 10 had a clinical examination and molecular testing but not a radiographic examination. All patients were short and were not able to stand or walk. To get around, they slid on their trunk and pelvis by pushing their hands against the ground. Sometimes, they use wheelchairs when available. Besides brittle bones, their general health was unremarkable, except for patient 3, who, according to his parents’ verbal report, defecated once every three months. All patients were cheerful with normal intelligence. The health of all parents was unremarkable.

**Figure 2:**
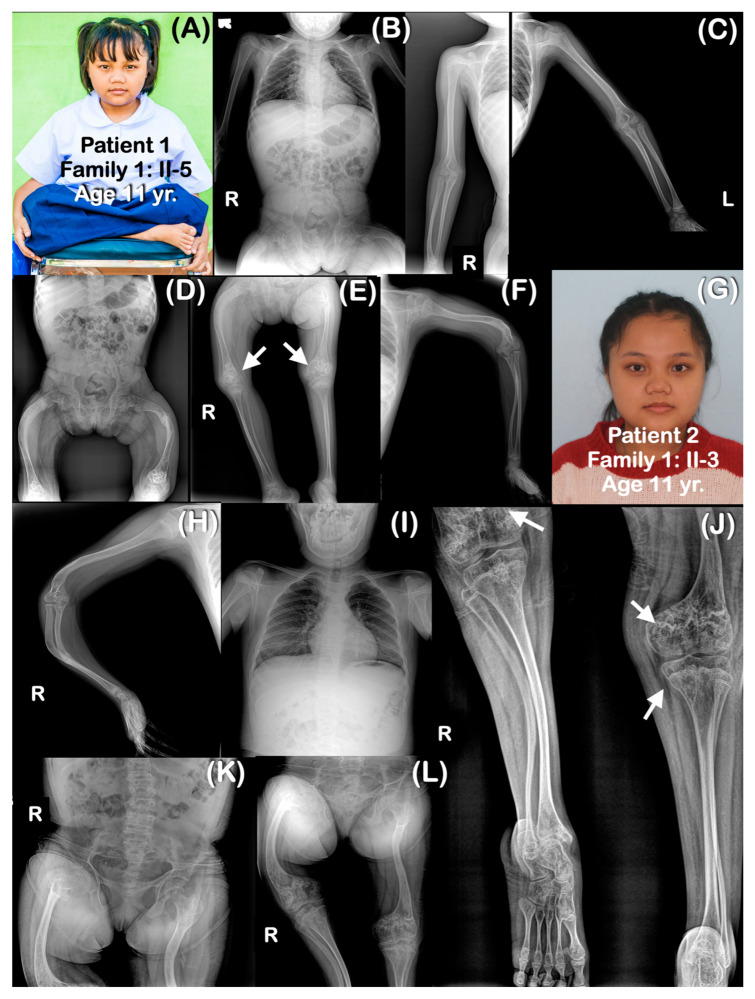
Family 1. (**A**–**F**) Patient 1 (Family 1: II-3). Age 11 years. Generalized osteopenia is noted. Ribbon rib deformity is observed. (**E**) Popcorn calcifications are observed at distal femoral metaphyses (arrows). Femora are shortened and curved. Fracture of left femur is healed. Humeri, radii, ulnae, tibiae, and fibulae are not bowed. (**G**–**L**) Patient 2 (Family 1: II-5). (**G**) Sister of patient 1. Age 17 years. Radiographs taken at 10 years. Generalized osteopenia is noted. Curved and slender long bones. The right femur is shorter than the left one. (**L**) Popcorn calcifications are observed at distal femora (arrows).

**Figure 3:**
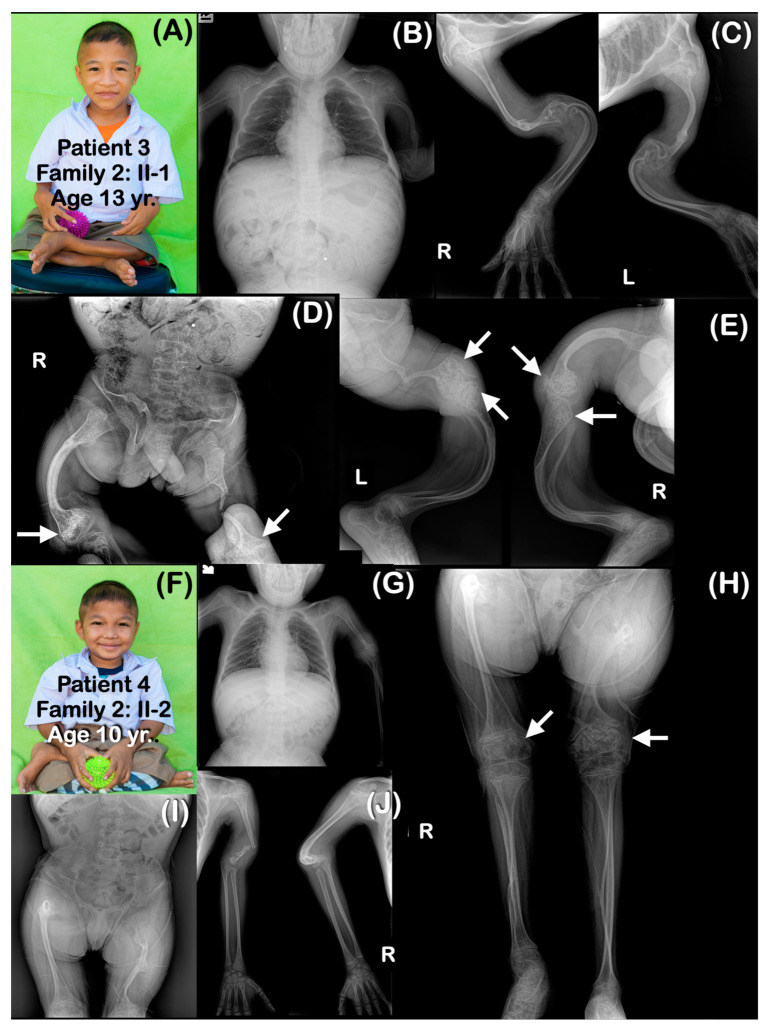
Family 2. (**A**–**E**) Patient 3 (Family 2: II-1). Age 13 years. Elder brother of patients 4 and 5. Generalized severe osteopenia. Severely dysplastic pelvis. The ribs are gracile (thin and slender). All long bones are severely slender and curved. Femora and humeri are shortened. (**D**,**E**) Popcorn epiphyses and metaphyses are observed at distal femora and proximal tibiae (arrows). Fractured humeri and right femur are healed. Left femur is severely shortened, and there is a fracture within proper healing in the middle part. (**F**–**J**) Patient 4 (Family 2: II-2). Age 10 years. Brother of patients 3 and 5. Generalized osteopenia. Severely dysplastic pelvis. All long bones are slender. Humeri and femora are shortened and curved. Fracture of left humerus is noted. (**H**) Large metaphyseal flare of distal femora and proximal tibiae is noted. Popcorn epiphyses and metaphyses are observed at distal femora (arrows).

**Figure 4:**
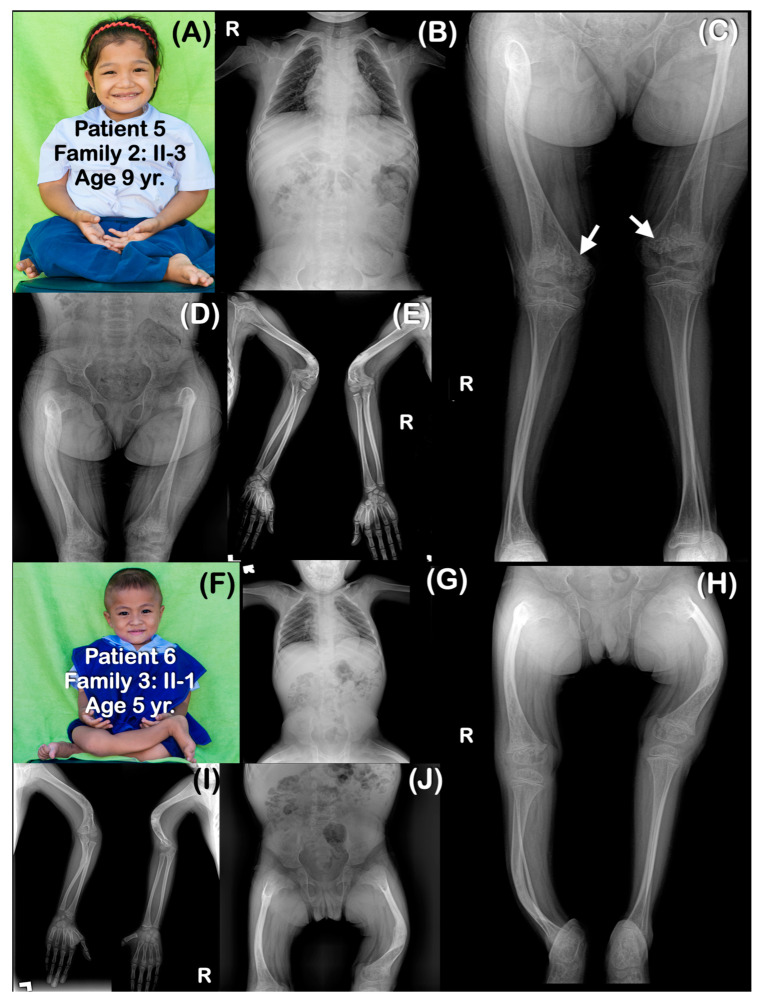
(**A**–**E**) Family 2. Patient 5 (Family 2: II-3). Age 9 years. The sister of patients 3 and 4. Generalized osteopenia. All long bones are slender. Humeri are shortened and sharply bent distally. Healed greenstick fractures of distal humeri are observed. (**C**) Large metaphyseal flare of femora is noted. Popcorn epiphyses and metaphyses are observed at distal femora (arrows). (**F**–**H**) Family 3. Patient 6 (Family 3: II-1). Age 5 years. Generalized osteopenia. Absence of trapezium, trapezoid, pisiform, and triquetrum bones bilaterally. All long bones are slender and bowed. Flare of distal femoral metaphyses is noted. Fractures of right humerus, bilateral humeri, and left femur are healed. Popcorn calcifications are not observed.

**Figure 5:**
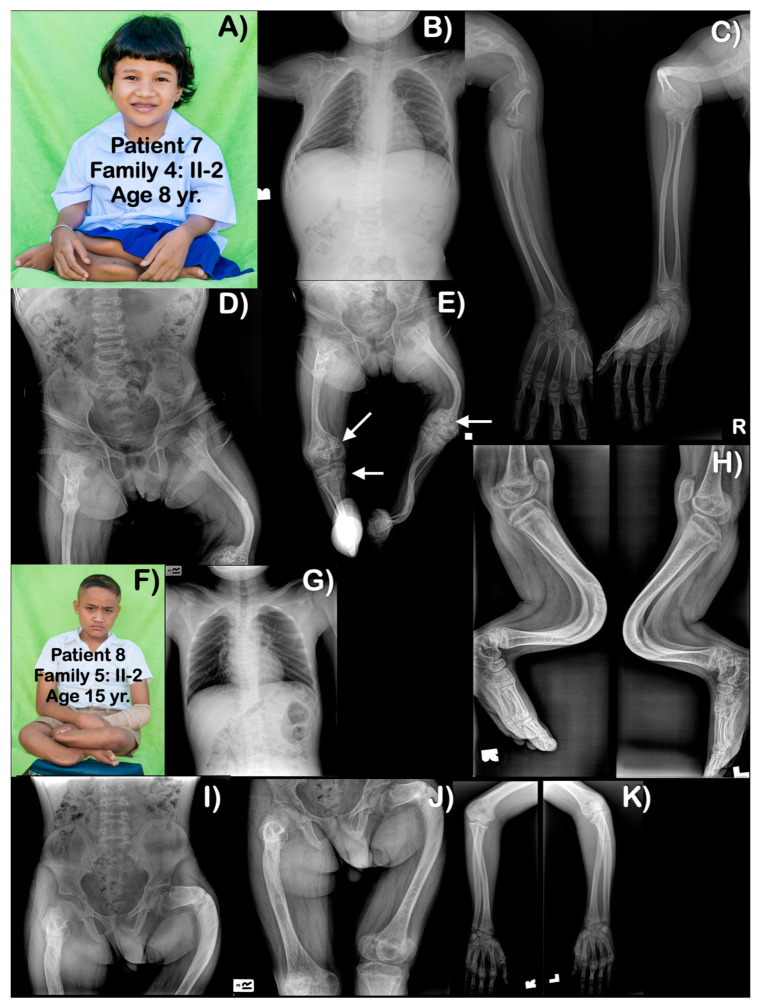
(**A**–**E**) Family 4. Patient 7 (Family 4: II-2). Age 8 years. Generalized osteopenia is evident. All long bones are slender, with humeri, femora, and tibiae severely shortened and curved. Proximal epiphyses and metaphyses of humeri are large. Multiple past fractures of long bones are evident. (**E**) Distal femora are bulbous with popcorn calcifications (arrows). Right proximal tibia has popcorn calcifications (arrow). (**F**–**K**) Family 5. Patient 8 (Family 5: II-2). Age 15 years. Generalized mild osteopenia. Humeri and femora are shortened. Fractured left proximal ulnar is seen. Unlike other patients, long bones are not slender. Femora and left humerus are bowed. Tibiae and fibulae are severely bowed anteriorly. Popcorn calcifications are not observed.

**Figure 6:**
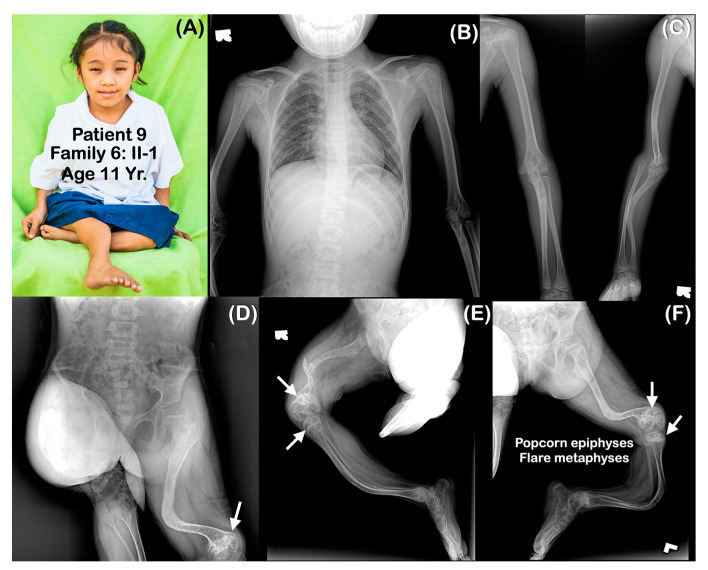
(**A**–**F**) Family 6. Patient 9 (Family 6: II-1). Age 11 years. Generalized osteopenia. All long bones are slender and curved. Femora are severely shortened. Fracture of right femur is noted. (**D**–**F**) Popcorn epiphyses and metaphyses at distal femora are visible (arrows).

**Figure 7:**
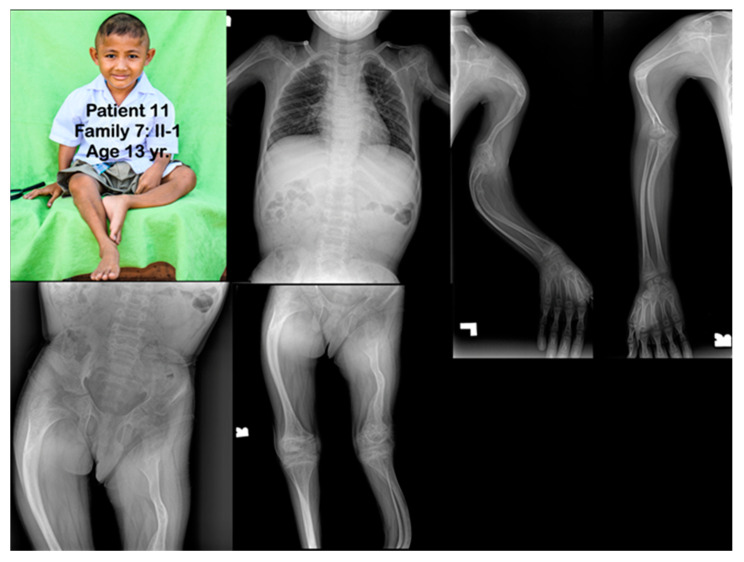
Family 7. Patient 11 (Family 7: II-1). Age 13 years. Generalized severe osteopenia. All long bones are severely slender and bowed. Humeri and femora are shortened. Fractures of clavicles are noted. Multiple fractures of long bones are healed. Metaphyseal flare of distal femora, proximal tibiae, proximal humeri, and distal humeri are noted. Popcorn calcifications are not observed.

All affected patients shared similar radiographic findings, including generalized osteopenia, a short thorax, platyspondyly, and slender ribs. Long bones were slender and curved with multiple fractures, resulting in severe bowing (Figure 2, Figure 3, Figure 4, Figure 5, Figure 6 and Figure 7). The metaphyses were bulbous, flared, and irregularly ossified. Phalanges appeared long. Some patients had curved humeri, curved tibiae, and fibulae, and some had straight bones. Shortening of the proximal segment of the long bones (rhizomelia) was observed in patients 3–7. Popcorn calcifications were observed in the distal femora and proximal tibiae of most patients (patients 1–5, 7, 9) (Table 1) (Figure 2, Figure 3, Figure 4, Figure 5, Figure 6 and Figure 7). For patient 10 (II-4) in family 6, only genetic testing was performed: the patient was not available for a radiographic examination. Patient 11 (family 7: II-1) had clinical and radiographic examinations, but not genetic testing. However, the father of patient 11 had genetic testing performed. Notably, none of the patients had ocular proptosis, which is a feature of patients with OI.

### 2.2. Whole-Exome Sequencing and Mutation Analysis

Genomic DNA was extracted from the saliva of 10 patients and their available family members according to the prepIT^®^ L2P protocol for the purification of genomic DNA using the Oragene^®^ collection kit (DNA Genotek Inc., Ottawa, ON, Canada)**.** Eight DNA samples from two affected patients (patients 1 and 2), four unaffected siblings, and their parents in family 1 were targeted for a whole-exome sequencing (WES) (Marcogen Inc., Seoul, Korea) protocol to detect variants in protein-coding genes.

**Figure 8:**
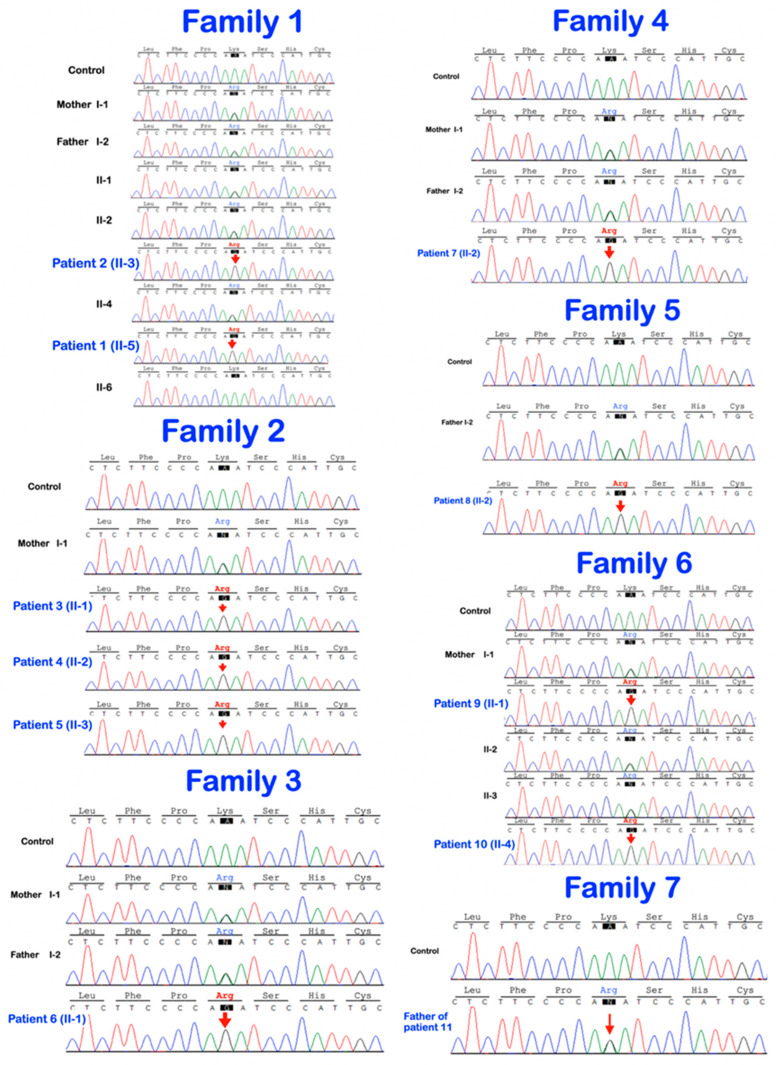
Sequence chromatograms show the homozygous and heterozygous base substitution (chr1:43212857A > G; NM_022356.4: c.2055 + 86A > G) in the *P3H1* gene. The patients are homozygous for the variant. The unaffected parents and unaffected siblings are heterozygous for the variant.

Exon capture was performed using the SureSelect target Enrichment system (Agilent, Santa Clara, CA, USA), and then pair-end sequencing was conducted on a HiSeq 2000/2500 sequencing machine. The Burrows-Wheeler Alignment tool (BWA-0.7.17) was used to align the 100 bp paired-end reads from the sequencer with the human reference genome assembly (hg19 from UCSC; GRCh37 from NCBI). Single-nucleotide variants (SNVs) and small INDEL variants were identified by Picard (picard-tool-2.9.0), Genome analysis toolkit (GATKv3.8.1), and Ensembl VEP (VEP bulid 105). Mutation discovery was based on the use of variants called from the WES of eight members of family 1. Bidirectional direct sequencing (Functional Biosciences, Madison, Wisconsin, USA) was carried out with DNA from all eight family members: two affected (II-3 and II-5) and four unaffected siblings (II-1, II-2, II-4, II-6) and their unaffected parents (I-1 and I-2). Sequencher 4.8 Sequence analysis software (Genecodes, Ann Arbor, MI, USA) was used to analyze the presence of variants and co-segregation between genotypes and phenotypes within family 1. Bioinformatic predictions of the variant impact were performed using MutationTaster (https://www.mutationtaster.org, accessed on 8 January 2023), PolyPhen-2 (http://genetics.bwh.harvard.edu/pph2, accessed on 8 January 2023), and SIFT (https://sift.bii.a-star.edu.sg/, accessed on 8 January 2023). Once the variant was identified in family 1, bidirectional direct Sanger sequencing of *P3H1* was performed with DNA from all 10 patients and their available family members (Figure 1 and Figure 8).

To investigate the possible effects of the variant on splicing, the contiguous sequence representing the last two exons and the final intron of *P3H1* were submitted as both the reference sequence format and the variant format to Alternative Splice Site Prediction (ASSP) [13].

## 3. Results

**Figure 9:**
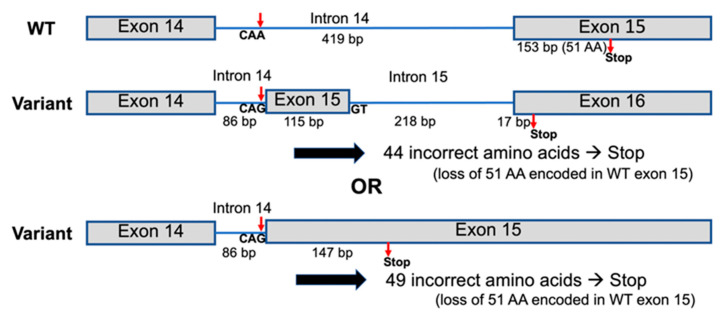
Two possible models for the effect of the *P3H1* variant on the splicing of P3H1 isoform a to form new isoforms. The variant CAG sequence is predicted to act as a constitutive splice acceptor site with 89% confidence, compared to an 8.6% chance for the consensus CAA sequence [13]. A GT sequence 116 bp later is predicted to act as a constitutive splice donor with 75% confidence. The use of this splice donor site gives the upper variant model an extra intron and exon and replaces the 51 amino acid residues encoded in exon 15 of isoform a with 44 alternative amino acids, with a frameshift in the final exon leading to an early stop codon. If the splice donor site is not used (lower variant model), intron 14 is reduced to 86 bp, and 49 codons are added from the former intron before a stop codon. In either case, the new C-terminal amino acid sequence is not functional.

Whole-exome sequencing and Sanger sequencing of all members of family 1 showed the homozygous base substitution chr1:43212857A > G (NM_022356.4:c.2055 + 86A > G) in the *P3H1* gene in all affected members (II-3 and II-5) and heterozygosity for this variant in all unaffected members (I-1, I-2, II-1, II-2, II-4, II-6) of the family, respectively. The variant is extremely rare and is not reported in the exome data from the Genome Aggregation Database (gnomAD) or Leiden Open Variation Database (LOVD). Sanger sequencing of all members of families 2–7 and the unaffected father of patient 11 confirmed the variant status in the available affected and unaffected family members (Figure 8). The variant creates a “CAG” trinucleotide following a poly-pyrimidine tract, which is typical of a splice acceptor site. To test this, the sequence containing the variant was evaluated using the splice site prediction algorithm, ASSP [13]. This analysis supported the conclusion that this new sequence was likely to act as a constitutive splice acceptor sequence, with 89% confidence. This level of confidence is higher than the normal splice acceptor site at the end of this intron, which has 78% confidence. A strong constitutive splice donor site (74% probable) is found 116 bp downstream from the mutation site. If utilized, it is predicted that this variant would lead to the incorporation of an alternative exon, which, when translated, would incorporate 37 novel amino acids and result in a frameshift with termination 6 codons into the last exon (Figure 9). If the strong constitutive splice donor site is not used and there is read-through, then it is predicted that a total of 49 novel amino acids would be added before termination (Figure 9, lower variant model). Both variant models result in the loss of the normal 51 amino acid residues from the C-terminus, including the KDEL sequence.

## 4. Discussion

We report on 11 patients from seven tribal Karen families affected by OI type VIII. All of the patients had generalized osteopenia, slender long bones, a history of multiple bone fractures, rhizomelia, the absence of blue sclerae, and platyspondyly (Table 1). They all lived in the same village at the border between Thailand and Myanmar. The sharing of the same variant (chr1:43212857A > G; NM_022356.4: c.2055 + 86A > G) in the *P3H1* gene implies a founder effect. This genetic variant has been reported in four unrelated Karen children affected by OI type VIII with dental anomalies. However, it was considered an exonic variant in isoform c [11]. Unfortunately, a dental examination was not performed in our study.

### 4.1. P3H1 and Its Pathogenic Variants

Prolyl-3-hydroxylase-1 is known to be crucial for bone development [1,14]. Specifically, prolyl-3-hydroxylation of α1(I) Pro986 is considered the most important post-translation hydroxylation of collagen, being crucial for protein–protein interactions, collagen folding, and the supramolecular assembly of collagen fibrils [3,6,8,15]. Prolyl-3-hydroxylase-1, encoded by the *P3H1* gene, is an active component of the 3-hydroxylation complex P3H1. Therefore, patients with abnormal P3H1 will not have an active prolyl 3-hydroxylation complex, resulting in the absence of α1(I) proline-986 hydroxylation. The presence of N-glycosylation and GAG attachment sites in prolyl-3-hydroxylase-1 implies that it also functions as an extracellular matrix proteoglycan. Its RGD cell attachment sites suggest a role in cell–matrix interactions.

It has been demonstrated that only the P3H1a isoform, which consists of 736 amino acid residues, is likely functional with respect to collagen modification. Unlike isoform b and isoform c, isoform a is the only isoform that has the Lys-Asp-Glu-Leu (“KDEL”) endoplasmic reticulum retention signal at the carboxy-terminus [12]. The “AA” to “AG” variant (chr1:43212857A > G; NM_022356.4: c.2055 + 86A > G) is predicted to form a new high-confidence constitutive splice donor, resulting in the incorporation of an incorrect amino acid sequence, premature protein truncation, and the subsequent removal of the C-terminus containing the KDEL signal. We predict that the loss of the KDEL sequence would mean a non-functional P3H1 isoform a (Figure 9), as it would not be retained in the endoplasmic reticulum where it would normally hydroxylate collagen.

Unfortunately, due to the remote location of the patients and their parents’ protective nature, we were not able to obtain fibroblasts or other cells from the patients in order to isolate RNA to directly test this hypothesis. If cells or RNA were available, reverse transcription PCR spanning exons 14 and 15 would have been possible, and therefore, we would have been able to validate any aberrant transcripts, including the size and nature of the changes in *P3H1* mRNA [12]. It would also have been possible to determine whether the two other isoform transcripts were still produced. Quantitative RT-PCR might also have been used to determine the relative amounts of transcripts through amplification with transcript-specific primers and Western blotting could be used to determine the presence of the P3H1 protein [12].

*P3H1* variants are associated with OI type VIII, a severe or lethal form of OI, in which several dysregulated protein interactions may be involved. The absence of P3H1 in mice causes delayed collagen secretion, the over-modification of collagen molecules, and subsequent abnormal extracellular matrices in collagen-rich tissues, such as bone, tendons, and skin [16]. Collagens from patients affected by pathogenic variants in *P3H1* have decreased 3-hydroxylation of α1(I) Pro986, resulting in the abnormal folding of the triple-helical region of type I α1 procollagen chains, the prolonged exposure of triple-helical domains to hydroxylation enzymes, and subsequent increased lysyl hydroxylation and glycosylation along the collagen helix. The secretion of collagen is delayed; however, total collagen secretion is increased [4,7].

The expression of P3H1 also requires the presence of the protein CRTAP [17]. Prolyl-3-hydroxylase-1 is mutually stabilized with CRTAP in the endoplasmic reticulum, and the absence of either one will lead to the degradation of the other. Loss-of-function or hypomorphic mutations in either *P3H1* or *CRTAP* have been shown to cause the loss of both proteins in the cells and the loss of the hydroxylation complex and its associated functions, leading to severe bone and cartilage phenotypes in patients. Since P3H1 forms a complex with CRTAP and PPIB, the phenotypes of patients with pathogenic variants in *CRTAP* are similar to those with *P3H1* pathogenic variants [18,19].

### 4.2. Phenotypic Variability

Scleral whiteness, neonatal fractures, broad undertabulated long bones, popcorn calcifications of distal femora and proximal tibiae, and shortening of the proximal segments of the limbs (rhizomelia), as observed in our patients, are common findings. Our patients, like most patients with *P3H1* pathogenic variants, had growth deficiency, a very low bone mineral density, and bulbous metaphyses [1]. However, intrafamilial variability is observed in the children of family 2. Patient 3 was more severely affected than his sister (patient 5) and his younger brother (patient 4). All three had popcorn calcifications. Like most previously reported patients with *P3H1* variants, blue sclera and ocular proptosis were not observed in any of our patients [12].

### 4.3. Popcorn Calcifications

Popcorn calcifications, areas of disorganized sclerotic lines in the metaphysis and epiphysis around the growth plate [20], were observed in the majority of our patients. They were found in the distal femoral regions and/or proximal tibiae of seven patients (patients 1–5, 7, 9) but were not observed in three other patients (patients 6, 9, 11). All three children in family 2 (patients 3–5) had popcorn calcifications. They have been suggested to be the result of the fragmentation and disordered maturation of the physis, which leads to coalesced osseous trabeculae or osseous trabecular aggregation [21]. Popcorn calcifications are not only found in patients with post-translational collagen modifications; they can also be found in patients with abnormal collagen gene-associated OI types III and IV. Popcorn calcifications are associated with growth deficiency, and unilateral popcorn calcifications contribute to leg length discrepancy [18,20]. It is interesting to note that in our patients and in previously reported patients, popcorn calcifications have been reported only at the distal femora and proximal tibia and disappeared as the patients aged [15]. Willaert et al. (2009) reported on the oldest-living patients affected by OI type VIII. Popcorn calcifications were observed, but subsequently, they disappeared and became more strikingly translucent at the age of 17 years [12]. Having popcorn calcifications in the distal femora and proximal tibia and their disappearance as patients age suggest a relationship with the growth rate of long bones. The distal femoral physis is the fastest-growing growth plate in the human body, increasing at a rate of 1.00 cm per year, producing 70% of the longitudinal growth of the femur and 40% of the overall growth of the lower extremity. The proximal tibia physis is the second-fastest growth plate [22]. It is hypothesized that whatever it is that controls the high differential growth of these two growth plates (production of high levels of cytokines etc.), it is possible that this growth generates an environment that allows the preferential production of popcorn calcifications.

### 4.4. Rhizomelia

The shortening of humeri and femora, as observed in our patients (patients 3–7, 11), is common in patients with *P3H1* pathogenic variants [12] and in *Crtap* and *Lepre1* (*P3h1*) KO mice [15,16], suggesting that they are negative consequences of the abnormal prolyl 3-hydroxylation complex. Rhizomelia, diaphyseal widening and bowing, and the widening and deformation of the epiphyses and metaphyses appear to be the result of the differential effects of an abnormal prolyl 3-hydroxylation complex on collagen formation and the growth of long bones that lead to the disorganization of growth plates [15,16] and abnormal bone remodeling [12].

### 4.5. Bowel Obstruction

Bowel constipation is quite common in patients with OI with pelvic deformity. In general, bowel constipation is associated with neurological impairments and confounding factors, such as physical immobility. In patients with OI, bowel constipation has an incidence ranging from 26 to 58% [23,24,25,26]. The bowel obstruction in patient 3 is likely the consequence of protrusio acetabuli (acetabular protrusion), which is the intrapelvic displacement of the acetabulum and femoral head [24,25,26]. Acetabular protrusion has been reported in 33–55% of patients affected by OI [26,27,28]. It leads to the narrowing of the pelvic outlet and results in a partial obstruction of the rectosigmoid colon [24].

## 5. Conclusions

Eleven tribal Karen children affected by OI type VIII are reported. Interfamilial and intrafamilial variability is demonstrated. All affected children carried a homozygous base substitution (chr1:43212857A > G; NM_022356.4:c.2055 + 86A > G) in the last intron of the *P3H1* gene. We show here that the variant likely creates a new putative “CAG” splice acceptor sequence, which is predicted to result in alternative splicing, premature protein truncation, and the subsequent production of non-functional P3H1. This variant appears to be specific to the Karen population. Our study emphasizes the importance of considering intronic variants.

## Figures and Tables

**Table 1:** Clinical, radiographic, and molecular findings in patients 1–11.

Families	Pt	Age (yr)	Mutations	Ability to Walk	Generalized Osteopenia	Slender Long Bones	History of Multiple Fractures	Platyspondyly	Blue Slcerae	Rhizomelia	Popcorn Calcification	Remarks
**Family 1**	**1**	11	chr1:43212857A>G (Homozygous)	Never	Yes	Yes	Yes	Yes	No	No	Yes	
	**2**	6	chr1:43212857A>G (Homozygous)	Never	Yes	Yes	Yes	Yes	No	No	Yes	
**Family 2**	**3**	13	chr1:43212857A>G (Homozygous)	Never	Yes	Yes	Yes	Yes	No	Yes	Yes	Bowel obstruction
	**4**	10	chr1:43212857A>G (Homozygous)	Never	Yes	Yes	Yes	Yes	No	Yes	Yes	
	**5**	**9**	chr1:43212857A>G (Homozygous)	Never	Yes	Yes	Yes	Yes	No	Yes	Yes	
**Family 3**	**6**	5	chr1:43212857A>G (Homozygous)	Never	Yes	Yes	Yes	Yes	No	Yes	No	Absence of some carpal bones
**Family 4**	**7**	8	chr1:43212857A>G (Homozygous)	Never	Yes	Yes	Yes	Yes	No	Yes	Yes	
**Family 5**	**8**	15	chr1:43212857A>G (Homozygous)	Never	Yes	Yes	Yes	Yes	No	No	No	
**Family 6**	**9**	11	chr1:43212857A>G (Homozygous)	Never	Yes	Yes	Yes	No	No	No	Yes	
	**10**	8	chr1:43212857A>G (Homozygous)	Never	N/A	N/A	Yes	N/A	N/A	N/A	N/A	
**Family 7**	**11**	13	N/A His father is heterozygous for the mutation.	Never	Yes	Yes	Yes	Yes	No	Yes	No	

## Data Availability

Not applicable.
